# Gallstone Disease and Its Correlation With Thyroid Disorders: A Narrative Review

**DOI:** 10.7759/cureus.45116

**Published:** 2023-09-12

**Authors:** Phanish Chandra Ravi, Thanmai Reddy Thugu, Jugraj Singh, Rachana Reddy Dasireddy, Sharanya Anil Kumar, Natasha Varghese Isaac, Abiodun Oladimeji, Victoria DeTrolio, Rasha Abdalla, Vineetha Mohan, Javed Iqbal

**Affiliations:** 1 Medicine and Surgery, SVS Medical College, Mahabubnagar, IND; 2 Internal Medicine, Sri Padmavathi Medical College for Women, Sri Venkateswara Institute of Medical Sciences (SVIMS), Tirupati, IND; 3 Internal Medicine, Punjab Institute of Medical Sciences, Jalandhar, IND; 4 Medicine and Surgery, AMA School of Medicine, Makati, PHL; 5 Medicine and Surgery, Vydehi Institute of Medical Sciences and Research Centre, Bengaluru, IND; 6 Medicine, St. John’s Medical College Hospital, Rajiv Gandhi University of Health Sciences (RGUHS), Bengaluru, IND; 7 Medicine and Surgery, Obafemi Awolowo University, Ile-Ife, NGA; 8 Medicine and Surgery, Jackson Memorial Hospital, Miami, USA; 9 Medicine and Surgery, Shendi University, Shendi, SDN; 10 Medicine and Surgery, Government Medical College Kottayam, Kottayam, IND; 11 Neurosurgery, Mayo Hospital, Lahore, PAK

**Keywords:** gallstones, hyperthyroidism, hypothyroidism, thyroid disorder, cholelithiasis

## Abstract

Over the years, several studies have revealed an important link between thyroid disorders and gallstone disease. According to these studies, hypothyroidism and hyperthyroidism are associated with cholesterol gallstone disease. This association between thyroid hormone disorders and cholesterol gallstone disease is due to the importance of thyroid hormones on cholesterol synthesis, bile functioning and content, and gallbladder motility. Several genes and receptors have been found on the thyroid gland, liver, and gallbladder to verify this association. These genes affect thyroid hormone secretion, lipid metabolism, and bile secretion. Defects in these various gene expression and protein functions lead to bile duct diseases. Other causes that lead to cholesterol gallstone disease are supersaturation of the bile with cholesterol and impaired gallbladder motility, which leads to bile stasis. This article has discussed these factors in detail while highlighting the association between thyroid hormones and cholesterol gallstone disease.

## Introduction and background

The correlation between thyroid disorders and gallstone disease has been the subject of extensive investigation across several generations. In particular, various hypotheses have been proposed to explore the potential relationship between thyroid disorders and the occurrence of gallstones. The prevalence rate of gallstone disease in general populations ranges from 10% to 15%, indicating its wide occurrence and impact on a significant portion of the population [1]. The prevalence of the illness is higher among females, elderly people, and individuals who possess specific risk factors like obesity, sudden weight reduction, and a physically inactive way of life [1].

A study on the association of thyroid hormone deficiency and chronic gallstone disease suggests that the increased hydrophobic character of biliary atresias (BAs) due to the diminished expression of hepatic detoxification enzymes promotes cholesterol crystal precipitation [2]. Another study suggests that both hyperthyroidism and hypothyroidism promote cholesterol gallstone formation [3].

Gallstone formation involves multiple factors, such as impaired bile flow, increased cholesterol saturation, and altered gallbladder motility. The pathogenesis of thyroid dysfunction in cholesterol gallstone formation varies between hyperthyroidism and hypothyroidism. Hyperthyroidism induces cholesterol gallstones by overexpressing hepatic nuclear receptor genes involved in cholesterol metabolism, while hypothyroidism promotes cholesterol gallstones through increased cholesterol biosynthesis [3].

The association between cholesterol gallstones and thyroid disorders is influenced by several factors, including the overexpression of hepatic nuclear receptor genes (LXRα, RXR) in hyperthyroidism and the promotion of cholesterol biosynthesis in hypothyroidism, both contributing to the development of gallstones in the biliary system [3]. In this review, we will focus on exploring the mechanisms underlying the association between gallstone disease and thyroid disorders, considering factors such as altered expression of hepatic detoxification enzymes, increased hydrophobicity of biliary bile acids, and the impact of thyroid hormone imbalance on cholesterol metabolism and biosynthesis.

## Review

Bile formation and composition

Bile is a body fluid produced by the liver that is essential for the excretion of cholesterol and toxins and also helps digest lipids from the small intestine [4]. Bile consists of around 95% water, in which several components are dissolved [5]. Hepatocytes produce primary bile in their canaliculi which is modified by cholangiocytes through secretory and reabsorptive processes [6]. Bile is made up of many elements, such as proteins, carbohydrates, lipids, vitamins, mineral salts, and trace elements. The major components of bile are bile acids, cholesterol, and phospholipids [7]. These major bile components are excreted in a relatively fixed proportion. However, stones can form if bile is supersaturated with one component [4]. 

Sex differences between the association of gallstone and thyroid disorders 

The relationship between gallstone disorders (GSD) and thyroid disorders associated with sex or gender is observed. In one of the studies, thyroid function parameters, total triiodothyronine (TT3), total thyroxine (TT4), and thyroid-stimulating hormone (TSH) were measured along with abdominal ultrasound for GSD diagnosis in euthyroid subjects. There was an inverse association between TT3/TT4 ratio and GSD in men. TT4 was an independent risk factor for GSD. These parameters were not significant in women. They also speculated that in women, the effect of thyroid function on GSD might be overshadowed by that of estrogen as it is a risk factor for GSD. It was concluded that low levels of TT3/TT4 ratio and high levels of TT4 were significantly and independently associated with GSD in euthyroid male subjects. There was no significant relationship observed in female subjects [8].

In another study, hypothyroid and euthyroid mice were studied with respect to the claudin-1 expression in the liver. Claudin-1 plays a role in bile secretion. In hypothyroid patients, bile excretion is decreased, which leads to an increased prevalence of GSD. It was found that in hypothyroid females, there was elevated claudin-1 expression, while reduced claudin-1 expression was found in hypothyroid males compared to the euthyroid animals [9].

Disturbing lipid homeostasis

The regulation of lipid metabolism is primarily governed by the thyroid hormone receptor (TR) and liver X-receptors (LXR) [10]. Cholesterol can activate LXR, while the binding of tri-iodothyronine (T3) to TR significantly influences the expression of genes dependent on thyroid hormone. Both receptors have the ability to form heterodimers with retinoid X-receptor (RXR), and therefore their signaling is also controlled by 9-cis retinoic acid [11,12,13]. The DIO2 gene is responsible for producing type 2 deiodinase (D2), which is an oxidoreductase selenoenzyme tightly regulated by the body. D2 plays a crucial role in activating thyroid hormone by converting thyroxine (T4) into T3, thereby producing a ligand that binds to the TR [14].

Regulation by liver x-receptor/retinoid x-receptor pathway

The LXR/farnesoid X Receptor (FXR)/Takeda-G-protein-receptor-5 (TGR5) system is critically involved in maintaining the balance of cholesterol and lipid levels within the body [15,16]. LXR and FXR work together to regulate the metabolism of cholesterol and bile acids. LXR is activated by oxysterols and controls genes related to the synthesis of fatty acids and triglycerides, such as SREBP-1c. FXR, on the other hand, is activated by the byproducts of cholesterol and bile acid clearance. Bile acids, through both FXR and TGR5, reduce triglyceride levels through various mechanisms, including suppressing the expression of SREBP-1c and influencing the uptake and breakdown of fatty acids through β-oxidation [15,16].

Although we have identified a considerable number of genes regulated by LXR, our understanding of genes suppressed by LXR is currently limited [17]. We are aware that the activation of LXR leads to the suppression of crucial hepatic enzymes involved in gluconeogenesis, such as phosphoenolpyruvate carboxykinase, fructose-1,6-bisphosphatase, and glucose-6-phosphatase [18]. As far as we know, the inhibition of the hDIO2 gene is the initial connection between T3 production and LXR/RXR signaling [10]. In summary, the hDIO2 promoter acts as a target gene for LXR/RXR, as it can be down-regulated by either 22(R)-OH-cholesterol or 9-cis RA [10].

Integration of cholesterol and triglyceride homeostasis by LXRs

Earlier research has demonstrated that LXRs function as detectors of sterols and restrict the accumulation of cholesterol by reducing its absorption while enhancing its reverse transport and breakdown [19]. Simultaneously, when the LXR pathway is activated by dietary cholesterol, it is anticipated to stimulate the synthesis of triglycerides in the liver and promote the storage of fat. This occurs through the upregulation of the metabolic cascade involving SREBP-1c, resembling the effects of insulin [20]. LXRs play a crucial role in both the baseline expression and the insulin-induced expression of SREBP-1c, as well as its downstream target genes [21,22]. LXRs have also been associated with hepatic carbohydrate metabolism as they stimulate the expression of genes involved in glucose storage while inhibiting gluconeogenesis. These actions align with their role in facilitating energy storage [23-25]. From an evolutionary perspective, LXRs have provided a solution to the significant metabolic challenge of restricting the buildup of cholesterol, which animals are capable of synthesizing internally, while simultaneously allowing the storage of fat as a high-energy fuel source [20].

Regulation by thyroid hormone and thyroid hormone receptors

Thyroid hormones are vital for maintaining proper body composition and balancing lipid levels within the body [26]. A recent discovery has highlighted the importance of local production of thyroid hormone through the conversion of T4 to T3 by D2 in thyroid hormone function [14]. Given the connection between thyroid hormone and fat metabolism, it is understandable that the local production of T3 by D2 can be influenced by factors involved in fat regulation. For instance, bile acids have been found to increase the expression of D2 in brown adipose tissue through TGR5 activation, resulting in resistance to obesity caused by a high-fat diet [27]. This indicates that pathways influenced by bile acids promote processes related to lipolysis, which includes the activation of D2 for T3 generation. Conversely, sterols through LXR signaling would support lipogenesis and the suppression of factors like D2 [10].

Elevated levels of cholesterol are observed in individuals with hypothyroidism and those with thyroid hormone resistance [28-30]. When individuals with hypothyroidism receive thyroid hormone treatment, their elevated serum cholesterol levels return to normal [28,31]. The liver maintains cholesterol balance through the coordinated regulation of three main pathways [32,33]. Two of these pathways ensure an adequate supply of cholesterol. One pathway is the de novo synthesis, primarily controlled by hepatic 3-hydroxy-3-methylglutaryl coenzyme A reductase (HMGC-R). The second pathway involves the uptake of cholesterol from the bloodstream through the low-density lipoprotein receptor (LDLR). The third pathway involves eliminating cholesterol by synthesizing bile acids, with the rate-limiting enzyme in this process being 7α-hydroxylase (CYP7A1) [34].

CYP7A1, also known as cholesterol 7α-hydroxylase, serves as the key enzyme that determines the rate of cholesterol conversion to bile acids and plays a central role in regulating cholesterol homeostasis [35,36]. It has been established that thyroid hormone enhances the expression of CYP7A1 mRNA, and this effect is of utmost importance for the cholesterol-lowering effects of thyroid hormone [35,36]. Thyroid hormone enhances the expression of HMGC-R, the crucial enzyme that limits the rate of cholesterol biosynthesis [37]. Thyroid hormone positively regulates the expression of LDLR, a receptor that facilitates the cellular uptake of LDL [38-40].

Induction of human cholesterol 7α-hydroxylase (CYP7a1)

The hepatic pathway responsible for bile acid synthesis is a highly coordinated metabolic process that plays a vital role in the absorption of dietary lipids and the regulation of serum cholesterol levels [41-43]. CYP7A1, the enzyme responsible for the crucial step in the classical bile acid synthesis pathway, is involved in regulating the rate of bile acid production. Notably, the activity of this enzyme shows a negative relationship with plasma low-density lipoprotein (LDL) cholesterol levels in both rodents and humans [44]. Conclusive evidence suggests that human CYP7A1 is indeed a genuine target gene of T3 in cultured human cells. To evaluate the impact of adenovirus TRβ1 expression on various crucial cholesterol metabolic genes, human liver primary cells and a liver cell line (HepaRG) were examined. In both of these cell types, T3 significantly stimulated the expression of human CYP7A1. Furthermore, the effects of T3 treatment on selected genes exhibited striking similarity between human and mouse primary cultures as well as native mouse liver [45]. The levels and activity of CYP7A1 have a significant impact on serum cholesterol levels [26,44,46]. In mice that lack functional LDL receptors, the reduction in serum LDL cholesterol levels through thyromimetic and T3-dependent mechanisms can be achieved. These effects strongly rely on the induction of CYP7A1 [47,48].

Thyromimetic effects on reverse cholesterol transport (RCT)

Thyromimetics are artificially created compounds that mimic the actions of thyroid hormones. These compounds are specifically designed to have a higher affinity for TRβ, primarily TRβ1, which plays significant roles in the liver for reducing serum cholesterol. This preference is in contrast to TRα1, which is associated with adverse effects of hyperthyroidism on the heart, muscles, and bones. Furthermore, thyromimetics are engineered to accumulate selectively in the liver, which is the primary site for regulating cholesterol metabolism [49]. Reverse cholesterol transport (RCT) is a multifaceted process that involves the transfer of cholesterol from peripheral cells to the liver for eventual removal in the feces as bile acids and neutral steroids. The concept of RCT was first introduced by Glomset over 40 years ago [50]. Research conducted on human hepatoma cells and primary human hepatocytes indicates that the expression and promoter activity of human CYP7A1 is actively suppressed in response to thyroid hormones. This suggests that THs and thyromimetic compounds would reduce the synthesis of bile acids [51,52].

Impact on canalicular transporters

The composition of biliary lipids in vivo is determined largely at the level of the hepatocyte canalicular membrane [53]. Bile formation is maintained by a network of ATP-binding cassette (ABC) transporters in the hepatocyte canalicular membrane, which regulates biliary regulation of bile salts, phospholipids, and cholesterol [53]. Defects in various ABC gene expression and protein function results in various cholestatic liver and bile duct disease [53]. Nuclear receptors such as FXR also regulate lipid transport proteins in the hepatocyte canalicular membrane and function as a bile salt receptor that regulates the transcription of numerous genes in maintaining cholesterol and bile salt homeostasis [53]. LXR, another subfamily of nuclear receptors, regulates the expression of ABCG5/G8 cholesterol transport protein, which is important in gallstone formation [33].

Thyroid hormones, on the other hand, are known to control essential functions in growth, differentiation, and metabolism [54]. Most of its gene regulation activities in the liver are explained by the interaction of the thyroid hormone receptor B1 (THRB1), the isoform present in the liver [55]. Administration of Thyroid hormone increases biliary cholesterol secretion and hepatic ABCG5/G8 expression levels, and the increase in cholesterol secretion was found to be predominantly exerted by ABCG5/G8 independent of LXRa [56]. T3 via THRB1 was also discovered to regulate ABCB4 protein levels at the canalicular membrane, thereby promoting phosphatidylcholine secretion into bile. This finding may be important in understanding Thyroid Hormone's role as a bile duct homeostasis regulator [57]. 

Gallbladder motility

One of the major causes of GSD is due to gallbladder motility dysfunction. This leads to bile stasis, either due to intrinsic defects in the gallbladder or due to impaired cholecystokinin (CCK) release [58]. CCK increases the contractility of the gallbladder. When CCK is impaired, this causes a disturbance in bile flow leading to bile stasis which in turn leads to GSD. Cholesterol hypersecretion causes gallbladder hypomotility and increases mucin secretion from the gallbladder epithelium. This, in turn, leads to the formation of biliary sludge, which contains glycoproteins and cholesterol crystals [59]. Cholesterol supersaturation in bile decreases the gall bladder's contractility, leading to bile stasis.

Impaired bile flow and viscosity

The rate of bile salt secretion is a major determinant of bile flow, and the secretion of biliary cholesterol and phospholipids is closely related to that of bile salts [60]. The study by Andreini et al. found that the administration of thyroid hormone to hypothyroid rats rapidly increased the release of cholesterol-rich vesicles in bile through a mechanism that included microtubules. This model supports the theory that biliary secretion of cholesterol and phospholipids may entail a particular vesicle secretory pathway by allowing observations during fast changes in biliary lipid secretion. Other effects of thyroid hormones on cholesterol metabolism may depend on T3's impact on biliary lipid secretion [60]. The effects of thyroidectomy and thyroid hormone therapy on the hepatic transport of endogenous bilirubin were studied. The hepatic bilirubin uridine 5'-diphospho-glucuronosyltransferase (UGT) activity was increased, and the p-nitrophenol transferase activity was decreased in hypothyroidism. It resulted in cholestatic conditions with a 50% reduction in bile flow and bile salt excretion, as well as an increase in the serum concentration of conjugated bilirubin [61]. Unconjugated and monoconjugated bilirubins' biliary output reduced concurrently by around 65%; however, the excretion rate of diconjugated bilirubin declined by only 47%, leading to an elevated di- to monoconjugate ratio in bile. The symptoms of hyperthyroidism were increased p-nitrophenol transferase activity, increased bilirubin production in bile, and decreased bilirubin levels. The excretion of the diconjugate increased by only 20% to 50%, depending on the amount of thyroxine given, whereas the production of unconjugated and monoconjugated bilirubin increased in parallel by about 50% or 0%. This led to a lower di- to monoconjugate ratio in bile.

A positive linear relationship between bilirubin UGT activity and the ratio of bilirubin di- to monoconjugates present in bile or formed by incubating liver homogenates in vitro at low concentrations of bilirubin (10 to 15 pM) was discovered, indicating that the conjugation activity in the liver primarily determines the makeup of bile pigment. Unconjugated bilirubin (UCB) and bilirubin monoconjugates (BMC) excretion rates reduced significantly and simultaneously in hypothyroidism, although bilirubin diconjugate (BDC) production decreased less noticeably. However, UCB and BMC excretion increased significantly, and simultaneously in hyperthyroidism, the increase in BDC production was far less prominent. Bile flow was found to be reduced by 54% in hypothyroidism. However, in the current study, hyperthyroid animals showed no alterations in bile flow [61]. Using quantitative cholescintigraphy, we formulated the conclusion that hypothyroidism may cause a delay in the biliary tract's emptying. Changes in biliary emptying could be one of the probable factors for the higher prevalence of common bile duct stone (CBDS) in hypothyroidism, in addition to the changes in bile composition and excretion rate that are thought to occur in hypothyroidism. The lack of the pro-relaxing action of thyroxine on the sphincter of Oddi, which we have previously demonstrated to exist ex vivo, maybe the explanation for this [62].

Sphincter of Oddi dysfunction 

The sphincter of Oddi (SO) may be the cause of the link between gallstone disease and thyroid dysfunction. The sphincter of Oddi (SO) motility regulates the flow of bile [63]. By virtue of regular bile flow, the impaired SO function raises the likelihood of producing CBDS [64].

Previous studies have shown that patients with CBD stones have a higher prevalence of being diagnosed with hypothyroidism than patients with gallbladder stones or age-, sex-, and hospital-admission-adjusted controls [63]. A study was conducted to investigate the function of thyroxine in SO by contrasting the functions of triiodothyronine (T3), progesterone, cortisone, estrogen, and testosterone [63]. The researchers come to the conclusion that whereas normal thyroxine concentrations do not affect the ex vivo non-specific KCl-induced SO contraction, they do decrease receptor-mediated acetylcholine and histamine. Triiodothyronine and thyroxine both have an inhibitory impact on SO; as a result, a thyroxine shortage might make the SO more tense since it loses its pro-relaxing properties [63].

Researchers also come to the conclusion that T4 actively encourages relaxation in human SO that expresses TR beta1 and beta2. A transcriptional mechanism is used to carry out this function, which needs the synthesis of fresh mRNA and proteins and ultimately causes the activation of K+ channels [64].

Rapid weight loss due to hyperthyroidism 

Weight loss can contribute to the occurrence of gallstones due to a potential mechanism in which bile with high cholesterol levels accumulates in the gallbladder and forms crystals [65]. When obese adults experience rapid weight loss through gastric bypass surgery or low-calorie diets, they become prone to forming gallstones [66]. According to Heida et al., among 288 obese children who underwent a 6-month lifestyle intervention program to induce weight loss, gallstones were observed in 17 cases (5.9%) [67]. An interesting finding from the study was that cholelithiasis (gallstone formation) was not observed in patients who lost less than 10% of their initial weight. However, among patients who lost more than 25% of their initial weight, one-quarter developed gallstones, which was significantly higher compared to other groups (p=0.028) [67]. A different study found that the risk of gallstone formation significantly increased when individuals experienced weight loss at a rate exceeding 1.5 kg per week [68]. This is suggestive that both significant and abrupt weight loss caused by hyperthyroidism can trigger the development of cholelithiasis (gallstones) [69].

It is widely recognized that thyroid dysfunction affects the composition and transportation of lipoproteins [70]. In hyperthyroidism, there is typically an increase in cholesterol excretion and turnover, resulting in decreased levels of serum LDL-cholesterol and high-density lipoprotein cholesterol [69]. Hyperthyroidism leads to elevated secretion of bile acids that carry an excessive cholesterol load, resulting in an increased transport of these bile acids to the gallbladder through the bile ducts [69]. Furthermore, rapid weight loss can act as a trigger for reduced gallbladder contractility due to a diminished response of the smooth muscle in the gallbladder to cholecystokinin [65]. The combination of heightened bile acid secretion from the liver to the gallbladder and impaired elimination of bile acids from the gallbladder to the duodenum can influence the crystallization of cholesterol-rich bile and contribute to the formation of debris within the gallbladder [69].

Impact on enterohepatic circulation and detoxification

According to research, an estimated 19% of cholecystectomy patients suffered from hyperthyroidism and 17% from hypothyroidism [71]. The intersectionality between the two conditions, cholesterol gallstone disease (CGD) and thyroid hormone pathologies, is based on the importance of thyroid hormones on cholesterol synthesis and degradation, biliary functioning and content, and gallbladder motility [71]. These two prevalent conditions can significantly influence enterohepatic circulation, which is the recycling of bile acids through the liver and intestines. CGD can lead to altered gallbladder functioning paired with bile acid formation, secretion, and absorption malfunction. To a greater extent, thyroid diseases such as hyperthyroidism and hypothyroidism fluctuate the proper functioning of metabolism, therefore leading to potential disruptions with bile acid enterohepatic circulation [71]. 

The process of enteric digestion brings bile acids and lipids to the ileum to be actively transported with sodium to be reabsorbed. The hepatic portion of digestion occurs in the liver sinusoid through sodium transporters [71]. In the event of excess bile acid and cholesterol, the overflow is sent back to the blood by special transporters such as MRP3 to be renally excreted [71]. Therefore, each aspect of this enterohepatic circulation that becomes dysregulated can lead to cholesterol gallstone formation. 

Studies have shown that patients affected with gallstones have modified bile acid formation, containing elevated levels of hydrophobic bile acids and a decreased number of hydrophilic bile acids [72]. This array of bile acid configurations can potentially lead to gallstone formation. Another aspect of CGD, impaired gallbladder emptying, can affect enterohepatic circulation by reducing bile acid secretion into the intestines [72]. Recent research by Portincasa et al. found that CGD patients had significant postprandial gallbladder emptying, which led to subsequent profound disruption in the reabsorption of bile acids in the ileum and decreased levels in the duodenum [72]. 

Moreover, another aspect to consider is the importance and role of circulation in the kidneys on cholesterol reabsorption. Hypothyroidism results in decreased renal plasma flow, decreased glomerular filtration rate, and impaired urine concentration and dilation [71]. Thyroid hormone has an impact on the excretion and reabsorption of endogenous substances, therefore becoming an important factor in cholesterol and lipid metabolism [71]. 

Furthermore, thyroid diseases, such as hypothyroidism, have been linked to alterations in bile metabolism. With reduced hepatic uptake and clearance of bile acids, a proper environment for the formation of CGD is ensured due to the increased number of circulating bile acids [73]. Additionally, studies have shown that thyroid hormones affect the expression of bile acid transporters in the liver [73]. This demonstrates the further influence of thyroid hormones on enterohepatic circulation and gallstone formation. A study by Song et al. that explored animals with hypothyroidism resulted in a significant depletion of bile acid transporter expression in the liver [73].

Further association of accumulation of bile acids and hepatocyte detoxification leads to CGD. The hepatocytes convert potentially toxic substances through detoxification [71]. Conjugated bile acids protect the liver from endogenous injury, however, in cholesterol diseases, there is a decrease in SULT2A1 gene expression, which would lead to hepatic injury, perhaps precipitating CGD [71]. Research has hypothesized that hypothyroid patients have decreased SULT2A1 expression, which has the potential to lead to CGD [71] ultimately.

Impact on nuclear receptor-mediated Lith gene expression

The fundamental cause of CGD is a disturbed equilibrium between cholesterol, bile salts, and phospholipids [53]. Genetic factors may play an important contributory role, and a 2- to 3-fold increased risk is observed among first-degree relatives and heritability estimates of 25%-29% [74,75].

The ABC transport proteins, which are expressed at the canalicular membrane and include ABCB4, the transporter for phosphatidylcholine [76], ABCB11, the bile salt export pump [77], ABCG5/ABCG8, which induce biliary cholesterol secretion [78], as well as the sterol regulatory enzyme (SRE), are the most important proteins in the hepatocyte that mediate lipid trafficking. Therefore, in this study, we look at several nuclear receptors' roles in gallstone formation.

Nuclear receptors: Nuclear receptors (NRs) such as metabolite- and hormone-sensing transcription factors bind to their DNA target sites as a monomer (steroidogenic factor, SF-1), homodimer (estrogen receptor, ESR), or heterodimer (FXR and LXR) before forming heterodimers with the retinoid X receptor (RXR) to change gene expression in response to dietary or endocrine signals [79]. Bile acids, phospholipids, steroid hormones, thyroid hormones, retinoids, and vitamin D are recognized endogenous ligands for NRs [80]. Numerous of these come from cholesterol [81]. The role of certain NRs, including heterodimeric FXR and LXR, and homodimeric ESR, in biliary lipid secretion and their possible therapeutic consequences for CGD are reviewed in this work.

Liver X receptor: The genes involved in sterol, bile acid, and lipid homeostasis are controlled by the LXRs, which include LXRα and LXRβ [82,83]. Additionally, they induce the development of cholesterol and phospholipid efflux transporters in the liver, such as canalicular ABCG5/ABCG8 [84] and ABCA1 [85], which were studied by Uppal et al. in transgenic mice with constitutively active LXR expression and fed lithogenic diets [86]. By giving LXR agonists to LXR transgenic animals fed a lithogenic diet, the canalicular transporters ABCG5/ABCG8, ABCA1, and CYP7A1 were activated in the liver. Increased mRNA levels of these genes are substantially linked with biliary cholesterol levels and saturation [87], indicating a potentially harmful function for LXR activation in human gallstone disease. A lithogenic (Lith) loci susceptibility map has been produced by analyzing gallstone characteristics in mouse strains [75,88-90]. In transgenic mice with intestinal expression of a constitutively active LXR, intestinal-specific LXR activation reduced cholesterol absorption [91]. This trait mediates cholesterol efflux and is linked with intestinal ABCG5/ABCG8 transporter overexpression [92]. Thus, intestine LXR activity would prevent CGD in contrast to hepatic LXR activation.

Farnesoid X receptor: FXRs induce the expression of ABCB11 [92,93]. ABCB4 and ABCB11 transporter expression is reduced in biliary phosphatidylcholine and bile salt secretion. Treatment of lithogenic-diet- fed gallstone-susceptible mice with FXR agonist GW4064 prevented cholesterol gallstone formation. It increased the expression of ABCB11 and ABCB4 transporters, resulting in higher bile salt and phospholipid bile concentrations in bile. Apart from its role in hepatic lipid homeostasis, FXR activity is also considered a regulator of lipid genes expressed in the gut. A subset of female gallstone patients shows a lower intestinal expression of FXR and its target genes, ileal lipid-binding protein (ILBP), and OSTα-OSTβ [94,95].

Estrogen receptor: ESR1 and ESR2 are two closely related classical homodimeric nuclear receptors that estrogens modulate to carry out their biological actions [96-98]. As a result, estrogens may increase the risk for CGD by improving the activity of the hepatic ESRs [99]. Even with high-cholesterol diets, high plasma levels of estrogens have been linked to increased activity of the enzyme HMG-CoA reductase, which controls the rate of cholesterol manufacture [100,101]. Wang et al. discovered that estrogens caused an increase in cholesterol production even in the presence of a high-cholesterol diet when studying AKR ovariectomized mice treated with estrogens and fed with high-cholesterol diets. SREBP2 expression increased in correlation with these modifications [101]. By boosting ABCG5/ABCG8 activity, estrogens may also affect the canalicular membrane [99].

Impact of thyroid hormone on nuclear-receptor mediated Lith gene (TR) expression

TRs come in two different isoforms, TR-α and TR-β (Nr1a1 and Nr1a2) respectively [102,103]. The two receptor systems exhibit similarities in molecular processes, target genes, and ligand-binding affinity, even though the LXRs and TRs belong to different receptor subgroups [104,105].

The mouse CYP7A1 gene promoter exhibits interactions between TR and LXR, indicating cross-talk between the receptors [106]. At the DR-4 location of the ATP-binding cassette transporter A1 gene promoter, TR and LXR compete for binding. These findings suggest that LXR and TR may interact during transcription [107].

T3 activates the gene promoter, as evidenced by the up-regulation of mouse LXR-mRNA and protein expression in the liver. Recent research has demonstrated that the human LXR-promoter is regulated by LXR α [108]. T3 also induces the human LXR-promoter in CV-1 cells, suggesting that thyroid hormone may increase the expression of LXR-mRNA, particularly in the liver of mammals [109].

Despite the claim that TR does not appear to alter the lipid metabolic cascade [110], several recent studies found that TR and LXR interact to maintain lipid homeostasis [107,111,112]. As a result, the discovery that T may control LXR-mRNA expression suggests that a cross-talk between TR and LXR might be a new therapeutic target for atherosclerosis and dyslipidemia. 

Impact of SREBPs in relation to thyroid hormone receptors on gallstone disease

Transcription factors include SREBPs. SREBP-1c predominantly but not exclusively increases the transcription of genes needed for fatty acid production [113]. In the presence of LXR agonists, LXRs bind to an LXR-binding site in the SREBP-1c promoter and activate SREBP-1c transcription [21,114]. Both thyroid hormone receptors (TR α, β) and LXRs bind to the DNA binding site direct repeat 4 (DR-4) and form heterodimers with RXR [106,115,116]. Viguerie et al. reported that SREBP-1c mRNA is down-regulated by T3 in human adipocytes, as shown by DNA microarray analysis [117]. Zhang et al. reported that T3 increases chicken SREBP-1 mRNA in chick embryo hepatocytes (CEH) under glucose administration [118]. Furthermore, in a recent report, Kawai et al. concluded that T3 induces human SREBP-1c mRNA in HepG2 cells derived from human hepatocytes [119].

At 28 loci, 32 gallstone disease association signals with genome-wide significance were identified using the additive model. Out of them, 11 are known variations, while 21 are novel variations. Through a recessive mode of inheritance, one of the novel common variations, rs708686 upstream of FUT6, dramatically increases its association with gallstone disease [120-122].

In summary, the largest gallstone disease (GWAS) to date discovered 21 novel gallstone disease variants. The connections found specifically highlight the intestinal compartment of enterohepatic circulation in the pathophysiology of gallstone disease and emphasize the importance of sequence variants in genes involved in cholesterol regulation. We conclude that sequence variations influencing the quantity of cholesterol secreted into bile or the ratio of cholesterol to bile acids will likely result in the formation of gallstones.

## Conclusions

The interplay of the various underlying mechanisms involved in the association between thyroid hormones and gallstone disease was explored in this article. The thyroid hormone's effect on nuclear factors and cholesterol transport proteins is key in cholesterol homeostasis and the development of gallstone disease. Hypothyroidism may cause common bile duct stones by causing a delay in biliary tract emptying and lack of pro-relaxing action on the sphincter of Oddi, while hyperthyroidism, on the other hand, leads to increased cholesterol turnover and consequently causes crystallization of cholesterol-rich bile and formation of debris within the gallbladder. Many studies are still ongoing to fully understand the complex interaction that brings about these associations. 
